# Plasmacytoid Urothelial Carcinoma of the Bladder Metastatic to the Stomach: A Case Report

**DOI:** 10.1155/2012/715951

**Published:** 2012-08-16

**Authors:** Philippe Nabbout, James Furr, Murugan Paari, Gennady Slobodov

**Affiliations:** ^1^Department of Urology, The University of Oklahoma Health Sciences Center, Oklahoma City, OK 73104, USA; ^2^Department of Pathology, The University of Oklahoma Health Sciences Center, Oklahoma City, OK 73104, USA

## Abstract

*Introduction*. Plasmacytoid urothelial carcinoma (PUC) of the bladder is a rare histological variant of urothelial carcinoma that was recently identified. Available data on this histological variant is limited. *Case Report*. We report the case of a 75-year-old man with presumed history of high-grade urothelial cancer of the bladder, treated with transurethral resection and Bacille Calmette-Guérin (BCG) in 2004. Six years after treatment of the bladder cancer, the patient underwent gastrectomy for an undifferentiated carcinoma of the stomach. On followup, patient developed right ureterohydronephrosis and peritoneal carcinomatosis. Biopsy of the bladder during stent placement revealed a plasmacytoid urothelial carcinoma of the bladder. Rereadings revealed that the initial bladder and gastric malignancies were also plasmacytoid carcinoma, indicating that, the patient had since 2004, a PUC of the bladder that spread to the stomach and peritoneal cavity. *Conclusion*. Plasmacytoid urothelial carcinoma of the bladder is an aggressive variant of urothelial carcinoma. Based on our case and the literature review, this tumor can be misdiagnosed because of its rarity, leading to treatment delays. Both the urologist and the pathologist need to have a high index of suspicion for PUC whenever they encounter unusual clinical and/or pathological findings.

## 1. Introduction

Plasmacytoid urothelial carcinoma (PUC) of the bladder is a rare histological variant of urothelial carcinoma that was recently identified. Data on this histological variant is limited. Less than 100 cases have been reported in the literature. Available data suggest that this type of tumor is aggressive and often diagnosed at an advanced stage. We report on a case of PUC that metastasized to the stomach and the peritoneum after being initially misdiagnosed eight years earlier. We then perform a brief literature review about this rare tumor.

## 2. Case Presentation

We report the case of a 75-year-old man with presumed history of high-grade urothelial cancer of the bladder, treated with transurethral resection and Bacille Calmette-Guérin (BCG) in 2004 by an outside urologist. The patient was followed up with regular cystoscopies and showed no evidence of recurrent disease. Then, six years after treatment of the bladder cancer, the patient presented with a partial small bowel obstruction and diffuse abdominal pain. Suspicious lesions within the stomach and duodenum were found on CAT scan at that time. Subsequent endoscopy showed an extensive, thickened lesion in the body and antrum of the stomach. Biopsy of this lesion was thought to be consistent with poorly differentiated gastric carcinoma. After receiving 6 cycles of neoadjuvant chemotherapy (epirubicin, oxaliplatin, capecitabine), the lesions responded well and the patient underwent a complete gastrectomy with jejunoesophageal anastomosis. Of note, peritoneal or hepatic metastatic disease were not detected on palpation during abdominal inspection. No evidence of malignancy was found on the gastric specimen removed at that time.

**Figure 1:**
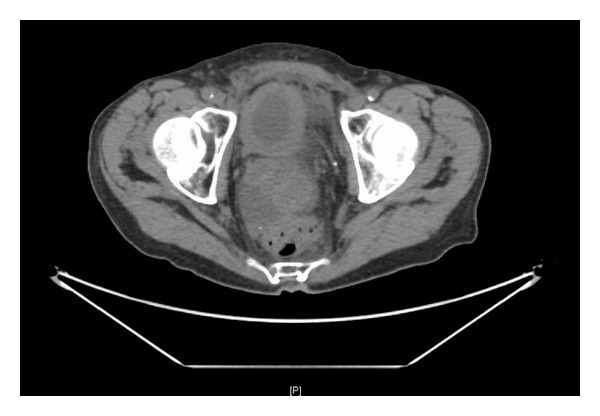
Pelvic CT showing diffuesly thickened bladder wall.

The patient was disease free on follow-up PET CT scans for approximately one year, at which time the patient began to have the flank pain, increased frequency, along with fatigue, weight loss, and diarrhea. Repeat PET CT scan at that time showed lesions in liver, peritoneal carcinomatosis, and right-sided hydronephrosis extending into the ureter down to the diffusely thickened bladder (Figure 1). At this point, the patient was referred to our urology department and subsequently underwent cystoscopy to investigate the right hydronephrosis.

Grossly, cystoscopy revealed a diffusely thickened and erythematous bladder wall with bullous lesions throughout and extensive mucosal oozing. Other than these bullous lesions, there was no apparent bladder tumor. The right ureteral orifice was highly stenotic and was the likely cause of the hydronephrosis. Random biopsies of the bladder were obtained, and a stent was placed on the right side. 

**Figure 2:**
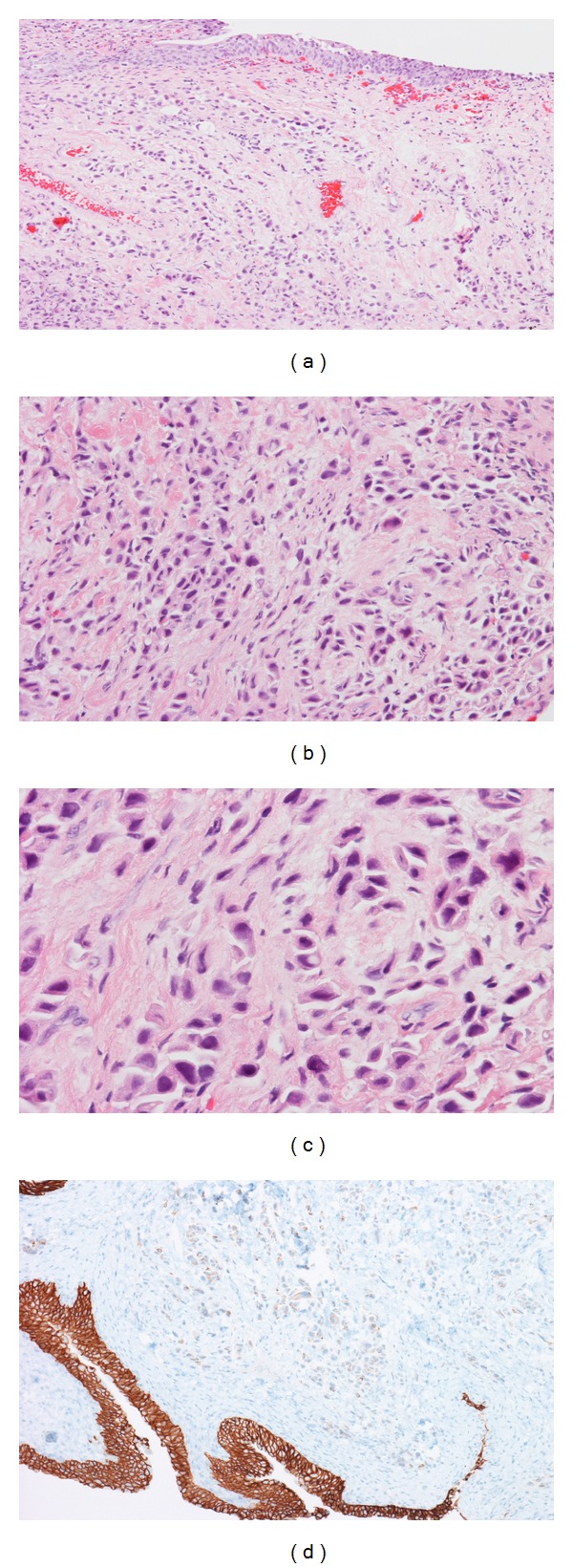
(a) Benign bladder mucosa (top) with diffusely infiltrating malignant cells in the lamina propria (Hematoxylin and eosin stain ×100). (b) Discohesive and loosely clustered malignant cells in an edematous stroma (Hematoxylin and eosin stain ×200). (c) The malignant cells closely resemble plasma cells with eccentric nuclei and moderate to abundant eosinophilic cytoplasms. Prominent retraction artefacts and few cells with cytoplasmic vacuoles (center) are also seen (Hematoxylin and eosin stain ×400). (d) E-Cadhedrin staining. Normally, the positivity is strong and diffuse like in the normal urothelial mucosa in the picture (bottom). It is markedly diminished in the tumor cells (upper part).

Biopsies revealed high-grade plasmacytoid malignant cells arranged in discohesive clusters. Prominent pericellular retraction artefacts and edematous stroma were also present (Figure 2). The cells were immunoreactive for CK 7, CK 20, MUC 1, and CD 138, and focally positive for intracytoplasmic mucin. E-Cadherin immunostain was markedly diminished (Figure 2). This staining pattern was consistent with the morphological diagnosis of a plasmacytoid urothelial carcinoma.

Suspicion was high that this patient's initial bladder cancer eight years prior was also a plasmacytoid variant; and that the gastric cancer, peritoneal carcinomatosis, and most recent bladder cancer were actually recurrences. A request was made for rereading of pathology slides from the initial bladder lesion in 2004, along with gastric biopsies from 2011. Rereadings revealed that the initial bladder and gastric malignancies were also plasmacytoid carcinoma, indicating that the patient had since 2004, a PUC of the bladder that spread to the stomach and peritoneal cavity. The patient has since been under the care of Hematology & Oncology service, and is currently receiving chemotherapy with gemcitabine and carboplatin.

## 3. Discussion

PUC is a rare and recently described variant of urothelial carcinoma which was included in the 2004 WHO classification for urothelial cancer [1]. Plasmacytoid morphology is typically found in lymphoma and plasmacytoma which led to the initial confusion in reporting these tumors. The first case of PUC was reported by Sahin et al. in 1991 [2]. Since then other case reports [3, 4] and series were published [5–10]. However, the majority of the series in the literature are small with the largest one having 32 patients [8]. Although limited, all available data show that PUC is usually a high-grade aggressive tumor, often with advanced stage at the time of diagnosis and poorer outcomes than conventional urothelial carcinoma of the bladder [5–10]. 

In our case the patient had peritoneal carcinomatosis and metastasis to the stomach. This pattern of spread is unusual and more aggressive when compared to conventional urothelial carcinoma of the bladder. In a recent review of 15 patients with PUC, Ricardo-Gonzalez et al. found that PUC had a predilection for intraperitoneal spread and can present with discontinuous involvement of serosal surfaces [5]. One of the explications for the aggressiveness of PUC and it's spread pattern is the loss of E-cadherin which is a protein necessary for cell-to-cell adhesion [11]. In fact, several authors [6, 8, 9] found that the majority of PUC are characterized by reduced expression or complete loss of E-cadherin and discohesive growth histologically. This is consistent with the pathology findings in our case. The discohesive growth pattern is also found in two other tumors characterized by loss of E-cadherin: lobular carcinoma of the breast and diffuse gastric carcinoma which should be in the differential diagnosis of PUC because they can spread to the bladder [12]. 

Another interesting finding in our case is that despite regular surveillance cystoscopies over a period of 6 years for the presumable TCC of the bladder, the outside urologist did not identify any tumor in the bladder. In fact PUC is often diagnosed late because of the absence of hematuria and lack of identifiable tumor in the bladder. Like our case, cystoscopy findings are usually indurated mucosa and thickened bladder wall [6, 9].

Because of the rarity of the disease, the treatment is not well defined. Since the majority of patients have advanced stage at diagnosis, treatment consists of radical cystectomy + adjuvant chemotherapy (cisplatin based) [3, 6]. Our patient already had metastatic disease by the time the correct diagnosis of PUC was made. However, an interesting finding is that the patient had a good response with the chemotherapy regimen that was given for the presumable gastric cancer. In fact the gastric and duodenal lesions regressed with the neoadjuvant chemotherapy before gastrectomy. More studies including larger number of patients are warranted to define treatment strategies for these patients.

Our patient had a significant delay in diagnosis of his PUC which potentially prevented him from receiving curative treatment before his disease became metastatic. We believe that this delay in diagnosis was the combined error of the pathologist and the urologist. We think that the pathologist should be aware about PUC and have it in their differential diagnosis whenever they have urothelial carcinoma with a discohesive growth pattern. The urologist should also have a high index of suspicion for PUC in every patient with a history of bladder cancer and suspicion for cancer involving peritoneal surfaces or intraperitoneal organs. In our case, a reread of the bladder pathology slides during the workup for the possible gastric cancer would have avoided the morbidity of the unnecessary gastrectomy.

## 4. Conclusion

Plasmacytoid urothelial carcinoma (PUC) of the bladder is a rare and aggressive variant of urothelial carcinoma. Based on our case and the literature review, this tumor can be misdiagnosed because of its rarity, leading to treatment delays. Both the urologist and the pathologist need to have a high index of suspicion for PUC whenever they encounter unusual clinical and/or pathological findings. More studies are needed to determine treatment strategies for this variant of urothelial carcinoma.
